# *PtomtAPX*, a mitochondrial ascorbate peroxidase, plays an important role in maintaining the redox balance of *Populus tomentosa* Carr

**DOI:** 10.1038/s41598-019-56148-w

**Published:** 2019-12-20

**Authors:** Bin Yin, Jiaxue Zhang, Yadi Liu, Xiang Pan, Zhijing Zhao, Hui Li, Chong Zhang, Conghui Li, Xihua Du, Yinjun Li, Di Liu, Hai Lu

**Affiliations:** 1 Beijing Advanced Innovation Center for Tree Breeding by Molecular Design, Beijing Forestry University, Beijing, 100083 People’s Republic of China; 2 College of Biological Sciences and Biotechnology, Beijing Forestry University, Beijing, 100083 People’s Republic of China

**Keywords:** Plant cell death, Plant molecular biology

## Abstract

Plant mitochondria are important energy-producing structure and ROS are generated as byproducts. APX is one enzyme of the AsA-GSH cycle to reduces H_2_O_2_ to water. We identified both *PtomtAPX* and *PtosAPX* are located in mitochondria of *Populus tomentosa* Carr. *PtomtAPX* is specifically targeted to mitochondria, while *PtosAPX* is dual targeted to both chloroplast and mitochondria. The expression of PtomtAPX in mitochondria was 60-fold that of PtosAPX by ELISA and qPCR analysis. Under high light stress, the expression levels of *PtosAPX* increased, while that of *PtomtAPX* only slightly changed. Compared to the WT, the antisense transgenic *PtomtAPX* cell lines showed slowed growth, smaller cells impaired mitochondria in MS medium under normal growth. RNA-seq results showed 3121 genes significantly altered expression in the antisense cells, and most of them are important for mitochondrial function, particularly in oxidative phosphorylation. Our findings demonstrates a mitochondrial location for one APX isoform, and provide valuable insight into the mechanism which ROS balance is modulated by AsA-GSH cycle in mitochondria.

## Introduction

Reactive oxygen species (ROS) are generated as byproducts of normal cell metabolism in several organelles and their production is enhanced under stress conditions^1^. APX is one enzyme of the ascorbate-glutathione cycle (AsA-GSH cycle) in plants^2,3^. APX in *Arabidposis* are localized to the cytosol (cAPX, AT1G07890, AT3G09640, AT4G32320), chloroplast (thylakoid-bound APX [tAPX, AT1G77490] and stromal APX [sAPX, AT4G08390]), microbody (including the peroxisome and glyoxisome) (mAPX, AT4G35000, AT4G35970) by organelle-specific targeting peptides and transmembrane domains^4–7^ and to remove H_2_O_2_ in the organelles themselves^2^. In Arabidopsis, *apx1*(AT1G07890) knockout plants showed increased H_2_O_2_ levels, higher sensitivity to oxidative stress, and suppressed growth and development^8–10^. *Arabidopsis apx3*(AT4G35000) knockout mutants did not show suppressed growth under normal or stress conditions^11^. *Arabidopsis* double *tapx*(AT1G77490)/*apx1* (AT1G07890) mutants showed late flowering, low protein oxidation during light stress and enhanced accumulation of anthocyanins^12^. The levels of ROS were increased and the germination was reduced in seeds of *Arabidopsis* APX6 knockout mutants^13^. Rice plants double silenced for cytosolic APXs showed normal growth and development and were able to survive under stress conditions^14,15^. Loss of function in OsAPX2(Os07 g0694700) showed semi-dwarf seedlings, yellow-green leaves and seed sterility^16^. Rice peroxisomal ascorbate peroxidase(OsAPX4; Os08g43560) knockdown showed early leaf senescence^17^. These results indicate that the APXs isoenzymes are indispensable for plant growth and development.

Jimenez *et al*. (1998) reported APX activity in the external side of the outer mitochondria membrane from pea^18^.Three different APX isoforms was showed in mitochondria of tomato (*Lycopersicon esculentum*) using native gel electrophoresis^19^. De Leonardis *et al*.^20^ reported high APX activity which maybe localized inside mitochondria using sonication-mediated disruption of potato mitochondria^20^. And the presence of more than one APX in mitochondria of both leaves and young green inflorescences of *Chenopodium album* was detected by native gel electrophoresis^21^. However, until now, no gene, cDNA, or protein sequence for the plant specifically mitochondrial isoform (mitAPX) has been described. *Arabidopsis* single or double chlAPX (sAPX and tAPX) mutants showed normal phenotype under normal growth conditions or under high light intensity stress growth conditions^22–24^. In addition, sAPX knockdown rice plants exhibit a normal phenotype and show normal biochemical and physiological performance under normal growth conditions^25^. These results suggest that sAPX is not important for H_2_O_2_ scavenging in chloroplasts and/or mitochondria of *A*. *thaliana* or rice.

In this study, we investigated there is a mitochondria-specific APX of *P*. *tomentosa* using green fluorescent protein (GFP) fusion experiments and immunoelectron microscopy. And *PtosAPX* is dual targeted to both chloroplast and mitochondria. The expression levels of *PtomtAPX* and *PtosAPX* were modulated by H_2_O_2_, NaCl, heat, drought, and cold. Compared to the WT, the antisense transgenic *PtomtAPX* cell lines showed slowed growth, smaller cells impaired mitochondria in MS medium under normal growth. The results indicated that *PtomtAPX* is specifically targeted to mitochondria and plays an important role in maintaining the redox balance in *Populus tomentosa* Carr.

## Results

### *PtomtAPX* is specifically targeted to mitochondria, and *PtosAPX* is targeted to both chloroplasts and mitochondria

The poplar database (the JGI Populus trichocarpav.1.1 genome browser; http://genome.jgi-psf.org/Poptr1_1/Poptr1_1.home.html; Tuskan *et al*. (2006)) was searched and 10 genes are predicted as APX proteins, and 2 genes(Protein Id: 209946, 798682) was speculated as putative mitochondrial/chloroplasts APX which has a mitochondria/chloroplast localization signal peptide. These homologous APX in *P. tomentosa* were cloned using primers specific for the *Populus trichocarpa* APX gene (Protein ID: 209946, 798682), respectivity. A 1,080 bp open reading frame (ORF) (homologous APX in *Populus trichocarpa*, Protein ID: 798682. Supplemental Fig. 1A) was isolated, sequenced, and named *PtomtAPX*, and a 1,086 bp ORF (homologous APX in *Populus trichocarpa*, Protein ID: 209946. Supplemental Fig. 1B) was isolated, sequenced, and named *PtosAPX*.

**Figure 1:**
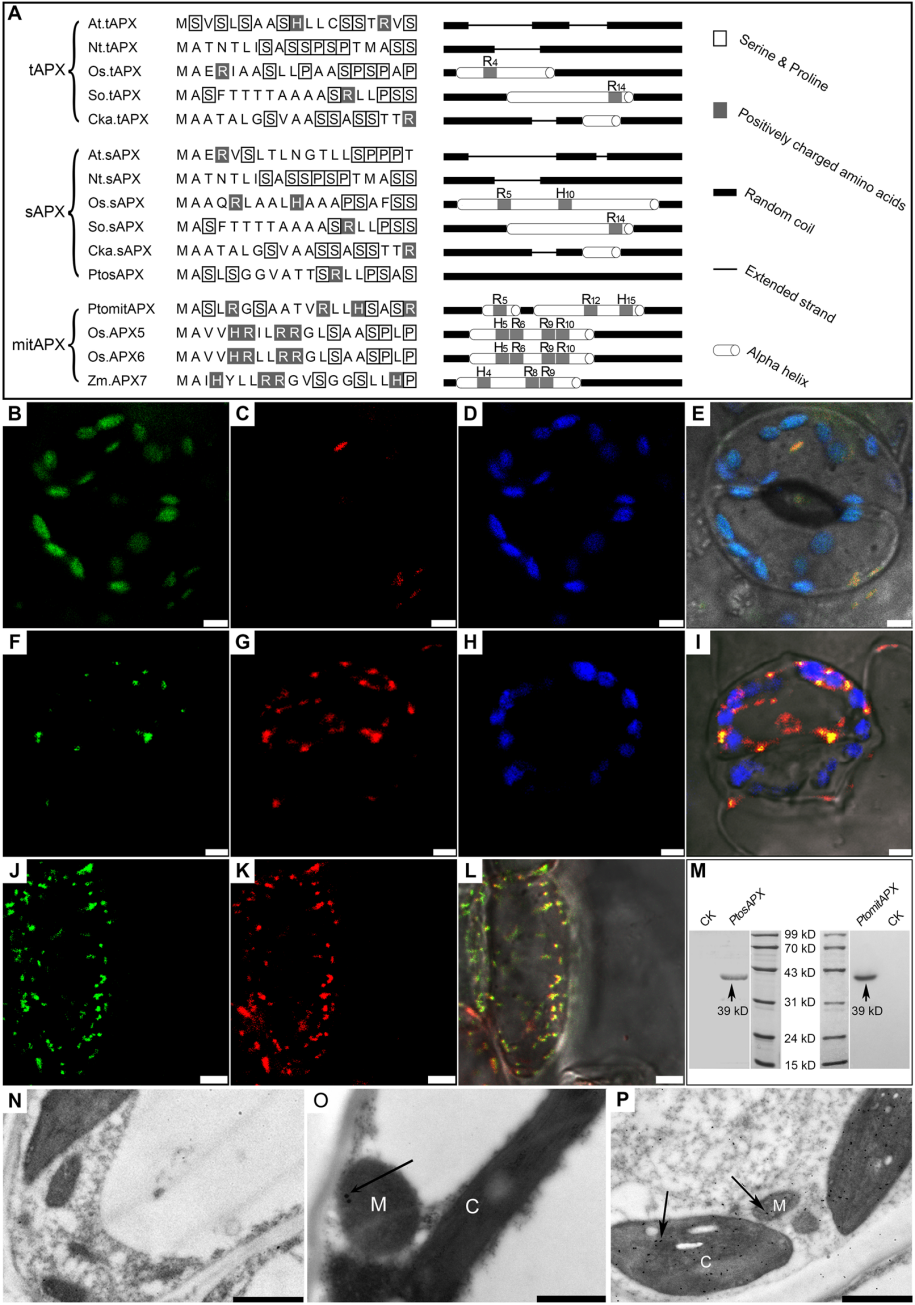
Amino acid sequence and subcellular distribution of *PtomtAPX* and *PtosAPX*. **(A)** Targeting peptide alignment and secondary structure analyses. **(B**–**E)** Expression of the PtosAPX-GFP fusion protein in leaf epidermal cells of transgenic tobacco. **(B)** Fluorescence of PtosAPX-GFP fusion protein. **(C)** Mitochondria stained with MitoTracker Red. **(D)** Chloroplast autofluorescence. **(E)** Merged image. **(F**–**L)** Expression of the PtomtAPX-GFP fusion protein in leaf epidermal and root-tip cells of transgenic tobacco. **(F**,**J)** Fluorescence of PtomtAPX-GFP fusion protein. **(G)** and **(K)** Mitochondria stained with MitoTracker Red. **(H)** Chloroplast autofluorescence. **(I**,**L)** Merged images. Bars, 5 µm. **(M)** Western blotting analyses of total protein extracts from leaf cells of *P*. *tomentosa* using anti-PtomtAPX and anti-PtosAPX antibodies. **(N)** Negative control. **(O)** Immunoelectron microscopy of *PtomtAPX*. **(P)** Immunoelectron microscopy of *PtosAPX*. 1 µm **(N**–**P)**. M, mitochondrion, C, chloroplast.

Sequence alignment revealed the presence of an N-terminal mitochondria/chloroplast-targeting peptide and two signatures of chloroplastic isoforms (KNIEEWP and ETKYTKDGPGAPGGQS) in *PtosAPX* and *PtomtAPX*, respectively (Fig. 1A and Supplemental Fig. 1). Phylogenetic analyses of APXs indicated that *PtomtAPX* and *PtosAPX* were chloroplastic and/or mitochondrial isoforms (Supplemental Fig. 2). Positively charged amino acid residues and amphipathic α-helix within the 19 N-terminal portion of the targeting peptide are important for the importation of proteins into mitochondria but not chloroplasts^26^. *PtomtAPX* had four positively charged residues and two amphiphilic α-helices in the targeting peptide, while *PtosAPX* had no positively charged residue and no amphiphilic α-helix (Fig. 1A). These results suggest that *PtomtAPX* is a mitochondrial isoform.

The subcellular localization of *PtosAPX* and *PtomtAPX* in *P*. *tomentosa* was determined by fusing their full-length coding sequences upstream of GFP under the control of the 35 S promoter (Fig. 1). The GFP signal of PtosAPX-GFP was detected not only in mitochondria co-stained with MitoTracker Red CMXRos (hereafter, CMXRos) but also in chloroplasts (red autofluorescence replaced by blue pseudocolor) in leaf epidermal cells. The GFP fluorescence of PtomtAPX-GFP was detected in mitochondria in leaf epidermal cells and root tips but not in chloroplasts.

Immunoelectron microscopy was performed to confirm the localization of *PtosAPX* and *PtomtAPX*. Immunogold labeling using rabbit anti-PtosAPX and anti-PtomtAPX antibodies showed that *PtosAPX* was localized to mitochondria and chloroplasts and *PtomtAPX* was localized to mitochondria (Fig. 1M–P). Taken together, these results suggest that *PtomtAPX* is localized to mitochondria in *P*. *tomentosa*, while *PtosAPX* is localized to both mitochondria and chloroplasts and shows greater similarity to a chloroplastic isoform.

### *PtomtAPX* and *PtosAPX* expression in mitochondria

**Figure 2:**
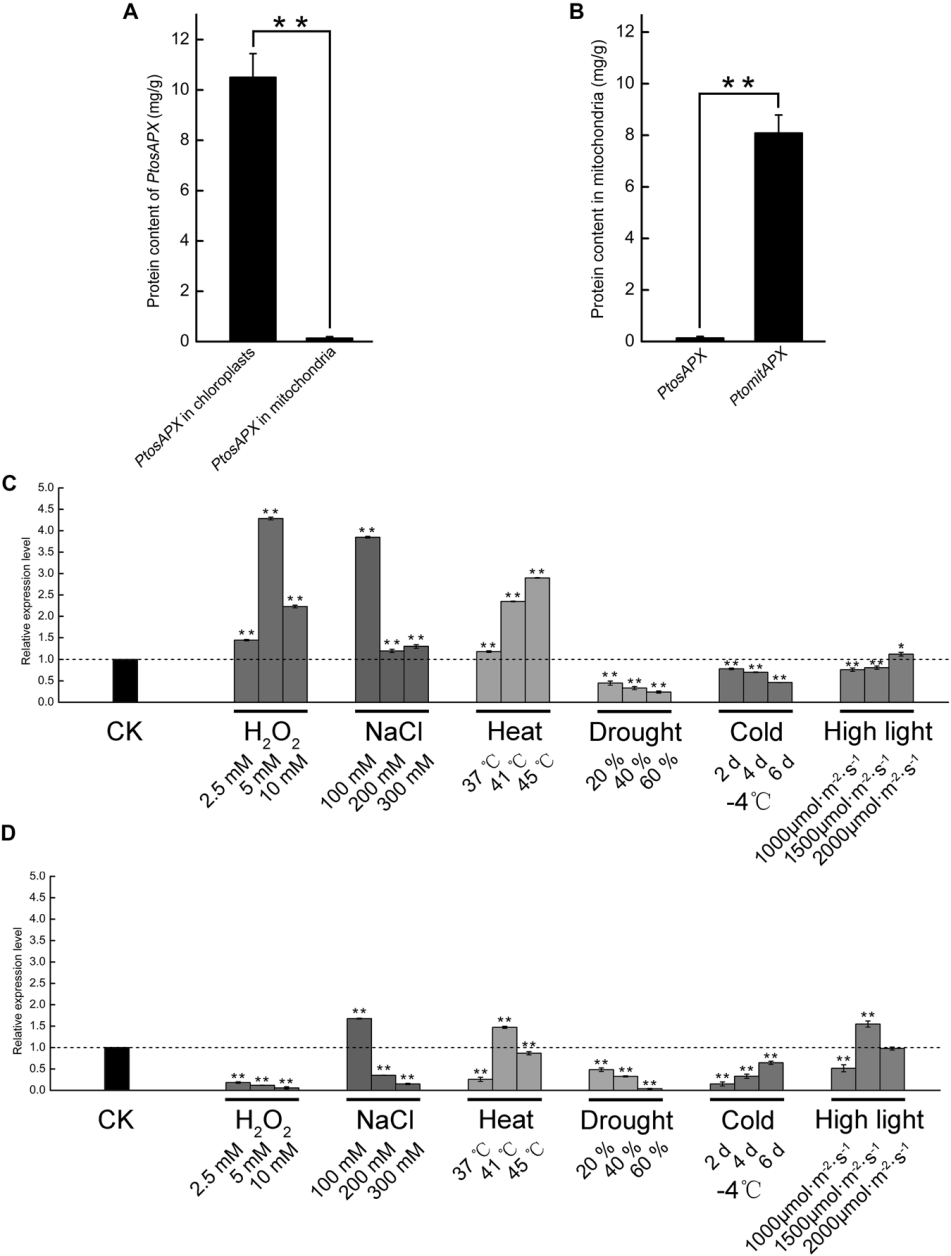
Expression profile of *PtosAPX* and *PtomtAPX*. **(A)** *PtosAPX* level in mitochondria and chloroplasts according to ELISA. **(B)** *PtosAPX* and *PtomtAPX* levels in mitochondria according to ELISA. **(C**–**E)** Expression of *PtomtAPX* and *PtosAPX* under abiotic stress according to qRT-PCR. **(C)** *PtomtAPX*. **(D)** *PtosAPX*. **Significantly different at P < 0.01 and *Significantly different at P < 0.05. Bars, standard deviations.

The presence of *PtomtAPX* and *PtosAPX* in mitochondria was investigated by enzyme-linked immunosorbent assay (ELISA). *PtosAPX* was detected in both mitochondria and chloroplasts. The *PtosAPX* level in chloroplasts was 78-fold that in mitochondria (Fig. 2A) and the *PtomtAPX* level in mitochondria was 60-fold that of *PtosAPX* (Fig. 2B). Therefore, *PtomtAPX* is the primary APX in mitochondria, while PtosAPX is likely to be a chloroplastic isoform.

The expression levels of *PtomtAPX* and *PtosAPX* in the presence of H_2_O_2_, NaCl, heat, drought, cold, or high-intensity light were determined by qRT-PCR with normalization to the actin gene (Potri.001G309500). Under high-light stress, the expression levels of *PtosAPX* increased and decreased, respectively, while that of *PtomtAPX* only slightly changed (Fig. 2C,D). The expression levels of *PtomtAPX* and *PtosAPX* were modulated by H_2_O_2_, NaCl, heat, drought, and cold.

### Enzymatic characteristics of *PtomtAPX* and *PtosAPX*

The expression constructs *pET30a-PtomtAPX* and *pET30a-PtosAPX*, which harbored the full-length cDNA minus the signal peptide, were expressed in *Escherichia coli*, resulting in production of recombinant *PtomtAPX* and *PtosAPX*, respectively. Sodium dodecyl sulfate (SDS)-PAGE showed that the molecular weight of purified recombinant *PtomtAPX* and *PtosAPX* were both 39 kDa. The reaction of *PtomtAPX* or *PtosAPX* with AsA and H_2_O_2_ followed Michaelis-Menten kinetics. At a fixed AsA concentration, the *K*_m_ and *V*_max_ values of *PtomtAPX* were 0.03 ± 0.00 mM and 1.54 ± 0.00 mM min^–1^ mg^–1^ for H_2_O_2_, respectively, while the *K*_m_ and *V*_max_ values of *PtosAPX* were 0.04 ± 0.01 mM and 1.64 ± 0.11 mmol∙min^−1^∙mg^−1^ for H_2_O_2_, respectively. At a fixed H_2_O_2_ concentration, the *K*_m_ and *V*_max_ values of *PtomtAPX* were 6.04 ± 0.06 mM and 3.00 ± 0.14 mM min^–1^ mg^–1^ for AsA, respectively, while the *K*_m_ and *V*_max_ values of *PtosAPX* were 4.30 ± 1.81 mM and 0.85 ± 0.25 mmol∙min^−1^∙mg^−1^ for AsA, respectively (Table 1). *PtomtAPX* and *PtosAPX* exhibited similar turnover rates (*k*_cat_), efficiencies (*k*_cat_/*K*_m_) and affinities (*K*_m_) for H_2_O_2_ and AsA. Therefore, *PtomtAPX* and *PtosAPX* are APX enzymes with similar activities.

**Table 1:** Enzymatic properties of recombinant PtomtAPX and PtosAPX.

APX isoforms	Substrate	*K*_m_ (mM)	*V*_max_ (mM min^–1^)	*k*_cat_ (min^–1^)	*k*_cat_/*K*_m_ (mM^–1^ min^–1^)
PtomtAPX	ASA	6.04 ± 0.06	3.00 ± 0.14	928405.63	153709.54
	H_2_O_2_	0.03 ± 0.00	1.54 ± 0.00	475149.03	15838330.11
PtosAPX	ASA	4.30 ± 1.81	0.85 ± 0.25	897148.78	208505.07
	H_2_O_2_	0.04 ± 0.01	1.64 ± 0.11	864716.73	20312959.95

Values are means ± SDs of three replicates.

### Decreased *PtomtAPX* content leads to mitochondrial dysfunction and PCD

**Figure 3:**
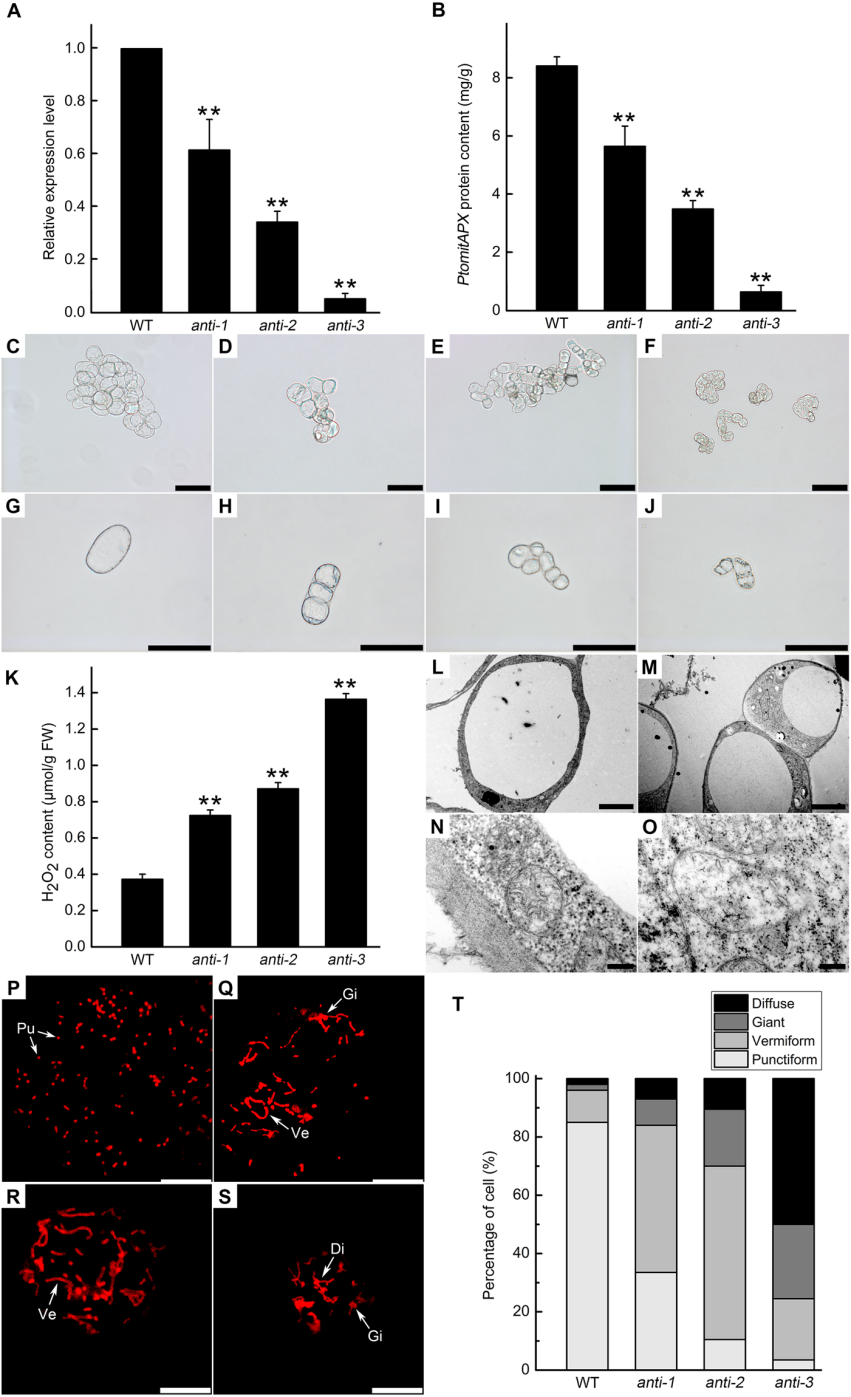
*PtomtAPX* expression, cell morphology and mitochondrial structure of *PtomtAPX*-antisense suspension cells. **(A)** qRT-PCR. **(B)** ELISA. **Significantly different at P < 0.01; bars represent standard deviations. **(C**–**J)** Cell morphology. **(C,G)** Morphology of WT; **(D,H)** *anti-1*; **(E,I)** *anti-2*, **(F,J)** *anti-3*. Bars, 100 μm. **(K)** Mitochondrial H_2_O_2_ content. **(L**–**O)** TEM of cell morphology and mitochondrial morphology. **(L,N)** WT; **(M,O)** *PtomtAPX*-antisense. Bars, 5 µm in **(L,M)** and 200 nm in **(N,O)**. **(P**–**T)** MitoTracker Red fluorescence of mitochondria in suspension cells of the WT **(P)**, *anti-1* **(Q)**, *anti-2* **(R)**, and *anti-3* **(S)**. Bars, 10 μm. **(T)** Frequencies of the types of mitochondria in 200 suspension cells. Pu, punctiform; Ve, vermiform; Gi, giant; Di, diffuse.

We used a pBI121-based construct to generate 45 antisense-*PtomtAPX* transgenic *P*. *tomentosa* suspension cell lines. The *PtomtAPX* transcript levels were 5–95% lower in 10 randomly selected *PtomtAPX* transgenic *P*. *tomentosa* cell lines compared to the WT. Three representative *PtomtAPX* transgenic cell lines with *PtomtAPX* transcript levels 61.63%, 34.29%, and 5.24% lower than the WT were named *anti-1*, *anti-2*, and *anti-3*, respectively (Fig. 3A). The *PtomtAPX* protein level was 67.18%, 41.55%, and 7.68% lower in *anti-1*, *anti-2*, and *anti-3*, respectively, than in the WT (Fig. 3B). Compared to the WT, the three transgenic *PtomtAPX* cell lines showed slowed growth and smaller cells in MS medium (Fig. 3C–J). Of the three cell lines, *anti-3* showed the smallest cells and the most seriously reduced growth (around 70%) compared to the WT. Compared to the WT, the mitochondrial H_2_O_2_ content was increased in *anti-1*, *anti-2*, and *anti-3*, respectively (Fig. 3K and Supplemental Fig. 3).

The structure of mitochondria in the *PtomtAPX*-antisense cells was assessed by transmission electron microscopy (TEM). WT cells exhibited a dense cytoplasm, clear structure, and a large vacuole occupying the majority of the cytoplasm (Fig. 3L). Numerous mitochondria with well-developed cristae and easily distinguishable outer and inner membranes were present in the WT cell lines (Fig. 3N). However, *anti-1*, *anti-2*, and *anti-3* cells harbored a high frequency of abnormal mitochondria, which lacked cristae and internal structures, but retained a double membrane (Fig. 3M,O). According to CLSM, the proportion of mitochondria with an abnormal morphology was significantly increased in the transgenic cell lines, particularly in *anti-3* (3.50% normal mitochondria compared to 85.00% in the WT) (Fig. 3P–T).

**Figure 4:**
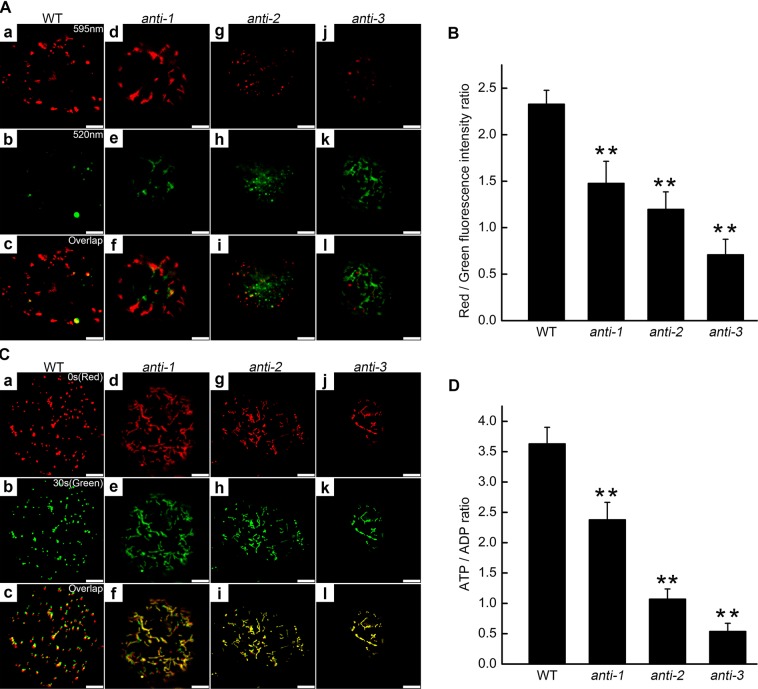
Mitochondrial Δψm in *PtomtAPX*-antisense suspension cells. **(A)** Δψm of mitochondria of suspension cells (JC-1 staining). Mitochondria with a high Δψm are shown in red (595 nm channel) **(**a), (d), (g) and (j); depolarized mitochondria are shown in green (520 nm channel); (b), (e), **(**h) and (k); (c), (f), (i) and (l), merged images; bars, 10 μm. **(B)** JC-1 red: green fluorescence ratios based on 200 cells. **(C)** CMXRos (red) fluorescence micrograph of mitochondrial mobility; green, pseudo-color; yellow, colocalized puncta; bars, 10 μm. **(D)** ATP:ADP ratios. Bars, standard deviations. **Significantly different at P < 0.01.

Changes in mitochondrial morphology may influence their function and decrease their ATP-generating capacity. To assess mitochondrial dysfunction, the mitochondrial membrane potential (Δψm) was determined using the fluorescent dye JC-1 and by calculating the red:green fluorescence ratio^27^.The function of mitochondria was impaired and the Δψm was markedly decreased in the *PtomtAPX*-antisense cell lines (Fig. 4A,B). Next, we visualized mitochondrial movement by CMXRos staining. Mitochondria were selected in time-lapse videos at 0 s (red images) and 30 s (green images) and the two images were overlaid; yellow mitochondria were considered non-motile. Mitochondria in WT cells showed motility, and few were yellow. By contrast, mitochondria in *anti-1* and *anti-2* cells showed reduced motility, while those in *anti-3* cells showed little motility (Fig. 4C). Therefore, mitochondrial motility was markedly decreased in the *PtomtAPX*-antisense cells, suggesting impairment of mitochondrial function.

Because ATP is produced in mitochondria by oxidative phosphorylation, and the decreased mitochondrial Δψm may be due to inhibition of electron transport chain (ETC) complexes, the ATP:ADP ratio and mitochondrial complex I, II, and III activities were determined. Compared to the WT, the ATP:ADP ratio was about 65.52%, 29.47%, 14.81%, and 21.27% in *anti-1*, *anti-2*, and *anti-3* cells, respectively (Fig. 4D). Compared to the WT, complex I, II, and III activities were significantly decreased in *anti-1*, *anti-2*, and *anti-3* cells, suggesting damage to ETC complexes (Table 2). Thus, energy production was decreased and ETC complexes were damaged in the *PtomtAPX*-antisense cells.

**Table 2:** Activity of respiratory complexes.

Group		nmol∙min^−1^∙mg^−1^ protein	
	Complex I	Complex II	Complex III
WT	93.59 ± 11.46	63.99 ± 8.36	99.28 ± 9.17
*anti-1*	75.71 ± 8.14	48.72 ± 8.99	81.17 ± 6.59
*anti-2*	58.60 ± 13.91	33.64 ± 7.09	52.75 ± 11.07
*anti-3*	31.71 ± 11.18	17.12 ± 6.95	25.32 ± 9.58
*OX*	91.16 ± 10.80	62.43 ± 8.12	100.17 ± 10.48

Values are means ± SDs of three replicates.

PCD is related to the overproduction of ROS in mitochondria^22,28,29^. Due to the reduced growth of antisense transgenic cell lines and plants, and the difficulty in obtaining the significant downregulation of the antisense plants, we suspected that deletion of *PtomtAPX* triggers PCD. We determined the degree of PCD in *PtomtAPX*-antisense suspension cell lines by two-channel flow cytometry (FCM). Normal (Q4, bottom left) and early apoptotic (Q3, bottom right) cells were stained by Hoechst dye, while apoptotic (Q1, top left) and advanced apoptotic (Q2, top right) cells were stained with propidium iodide (PI).

**Figure 5:**
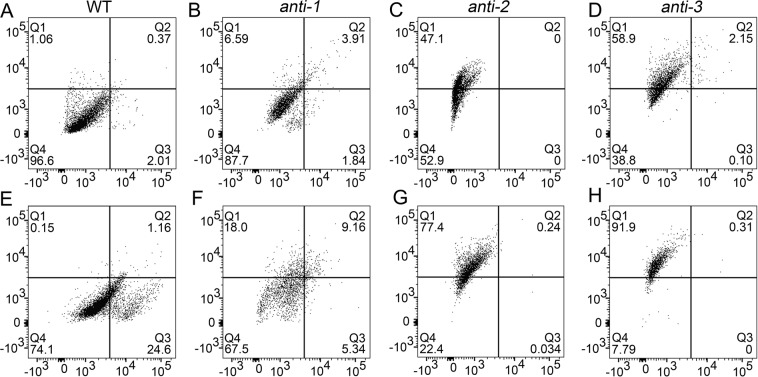
PCD of *PtomtAPX*-antisense suspension cells. **(A**,**D**,**G,J)** PCD under untreated conditions. **(B**,**E**,**H,K)** PCD under H_2_O_2_ treatment (10 mM, 3 h). Frequencies were calculated based on 5,000 cells per treatment.

In the WT, most cells were normal (>96%) and few were apoptotic (<2%) or dead (<2%). In comparison, the proportion of apoptotic *anti-1*, *anti-2*, and *anti-3* cells was increased by 12.30%, 47.10%, and 61.20%, respectively(Fig. 5A–D). Thus, the increased H_2_O_2_ content in the *PtomtAPX*-antisense cell lines resulted in a significant increase in PCD.

To further assess the relationship between H_2_O_2_ content and PCD, 10 mM exogenous H_2_O_2_ was added to the *PtomtAPX*-antisense cells to increase their H_2_O_2_ content. In the WT, adding 10 mM H_2_O_2_ increased the frequency of apoptotic cells by >25%, while the frequencies of apoptotic *anti-1*, *anti-2*, and *anti-3* cells were increased by about 32.50%, 77.60%, and 92.20%, respectively(Fig. 5E–H). Treatment with 10 mM exogenous H_2_O_2_ resulted in the death of almost all *anti-3* cells. Therefore, exogenous H_2_O_2_ increased the frequency of PCD in the *PtomtAPX*-antisense cell lines. Therefore, the H_2_O_2_ content is related to the degree of PCD.

The above results show that decreased *PtomtAPX* expression is correlated with increased PCD, which may explain both the reduced growth and difficulty generating, *PtomtAPX*-antisense *P*. *tomentosa* lines.

### Mitochondrial dysfunction is due to an increased H_2_O_2_ content and oxidative damage

Non-enzymatic antioxidants such as ascorbate (AsA) and glutathione (GSH) can reduce the increased H_2_O_2_ level in mitochondria or the cytosol^30^. To determine the role of AsA and GSH in removing H_2_O_2_, we determined the mitochondrial AsA, DHA, GSH, and GSSG contents of the *PtomtAPX*-antisense cell lines. Compared to the WT, the AsA contents were 52.07%, 37.43%, and 15.70%, and the AsA:DHA ratios were 48.56%, 34.08%, and 14.52% in mitochondria of *anti-1*, *anti-2*, and *anti-3* cells, respectively (Supplemental Fig. 4A). Compared to the WT, the GSH contents were 51.51%, 40.79%, and 27.37%, and the GSH:GSSG ratios were 43.40%, 36.01%, and 22.22%, in mitochondria of *anti-1*, *anti-2*, and *anti-3* cells, respectively (Supplemental Fig. 4B). The greatest decreases in the AsA:DHA and GSH:GSSG ratios occurred in *anti-3* cells. Thus, the transgenic cells are under oxidative stress and their H_2_O_2_ content could not be balanced by AsA and GSH.

An increased H_2_O_2_ content may lead to oxidative damage and reduce mitochondrial efficiency. Therefore, we evaluated mitochondrial lipid peroxidation by quantifying malondialdehyde (MDA) levels. Compared to the WT, the mitochondrial lipid peroxidation level was 1.74-, 2.55-, and 3.65-fold higher in *anti-1*, *anti-2*, and *anti-3*, respectively (Supplemental Fig. 4C). Moreover, the levels of oxidized proteins (with carbonyl groups) were increased in *anti-1*, *anti-2*, and *anti-3*, respectively, as determined by Western blotting (Supplemental Fig. 4D).

Therefore, the decreased *PtomtAPX* protein levels in the *PtomtAPX*-antisense cells resulted in increased H_2_O_2_ content, which could not be balanced by enzymatic or non-enzymatic antioxidant mechanisms due to the structural and functional impairment of mitochondria.

### Overexpression of *PtomtAPX* decreases H_2_O_2_ content and slows growth

**Figure 6:**
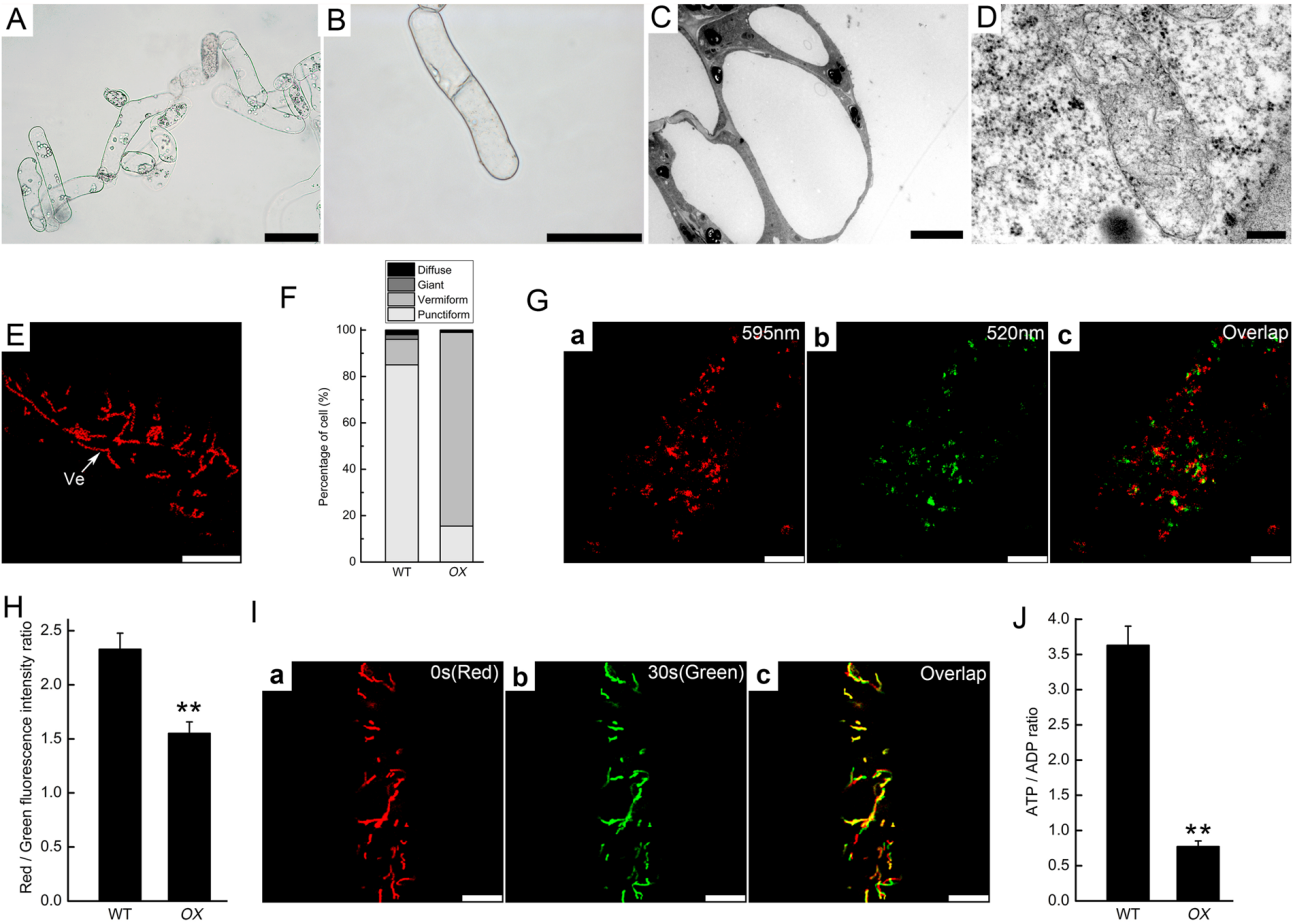
Growth reduction and mitochondrial dysfunction in *PtomtAPX*-overexpressed cells. **(A,B)** Morphology of *PtomtAPX*-overexpressed cells; bars, 100 μm. **(C**,**D)** Morphology of cells and mitochondria according to TEM; bars, 5 µm in **(C)** and 200 nm in **(D)**. **(E**,**F)** MitoTracker Red fluorescence of mitochondria; bars, 10 μm; Frequencies were calculated based on 200 cells. Ve, vermiform. **(G)** Δψm of mitochondria (JC-1 staining). **(H)** JC-1 polymer (red):monomer (green) fluorescence ratios based on 200 cells. WT, WT cells; *OX*, *PtomtAPX*-overexpressed cells. **(I)** Mitochondrial mobility; bars, 10 μm. **(J)** ATP:ADP ratios. **Significantly different at P < 0.01. Bars, standard deviations.

*PtomtAPX*-overexpressed cell lines were generated using a *PtomtAPX* overexpression construct under the control of the 35 S promoter (35Spro:*PtomtAPX*) (Fig. 6). qRT-PCR and ELISA showed that the *PtomtAPX* transcript and protein levels were increased 4-fold and 2.7-fold in the *PtomtAPX*-overexpressed cell lines compared to the WT, respectively.

For growth in MS medium, the *PtomtAPX*-overexpressed cell lines required 0.2 mg/L 6-BA. Compared to the WT, the *PtomtAPX*-overexpressed cell lines showed slowed growth and an abnormal morphology. The structure of mitochondria in *OX* cells was also abnormal by TEM and CLSM. The mitochondrial Δψm, motility, and energy production (ATP:ADP ratio) were decreased in the *PtomtAPX*-overexpressed plants, suggesting impairment of mitochondrial function. However, the complex I, II, and III activities were unchanged in *OX* (Table 2), suggesting that the respiratory chain complexes were not damaged.

The mitochondrial H_2_O_2_, AsA and GSH contents and AsA/DHA and GSH/GSSG ratios of the *PtomtAPX*-overexpression cells were not significantly different from those of the WT (Supplemental Figs. 4 and 5). The mitochondrial MDA level in *PtomtAPX*-overexpression cells was 41.02% of the WT (Supplemental Fig. 5). The oxidized protein level in *PtomtAPX*-overexpressed cells was slightly lower than that of the WT (Supplemental Fig. 4). These results suggest that oxidative damage was alleviated in the *PtomtAPX*-overexpression cells compared to the WT.

We suspected that the decreased H_2_O_2_ content in *OX* caused mitochondrial dysfunction, because H_2_O_2_ is an important signaling molecule. To further assess the relationship between H_2_O_2_ content and mitochondrial dysfunction, exogenous H_2_O_2_ was added to *PtomtAPX*-overexpressed cells. Addition of 10 or 100 mM exogenous H_2_O_2_ to *PtomtAPX*-overexpressed cells significantly alleviated mitochondrial damage and partially restored the mitochondrial membrane potential (Supplemental Fig. 5). Therefore, overexpression of *PtomtAPX* resulted in a decreased H_2_O_2_ content and mitochondrial dysfunction.

### Gene expression in *PtomtAPX*-antisense and *PtomtAPX*-overexpressed cell lines

**Figure 7:**
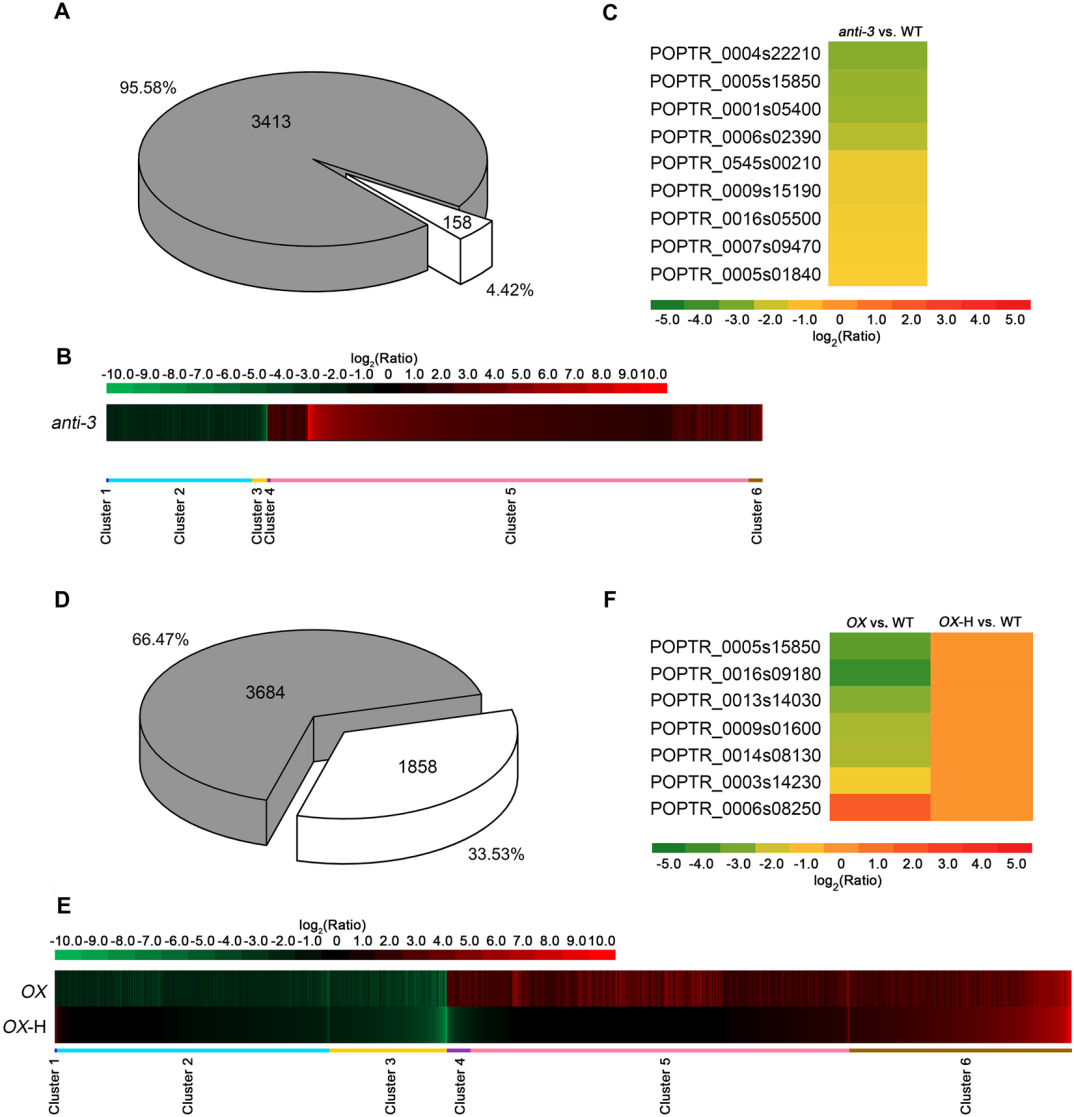
RNA-seq analyses of *anti-3* and *OX*. **(A)** DEGs of anti-3 (anti-3 vs. WT) and their expression under AsA treatment (1 mM, 3 h) (corrected P-value < 0.001, fold change >1.5 or < –1.5; gray, genes recovered to normal expression level under AsA treatment). **(B)** Clustering of expression ratios (*anti-3* vs. WT). Red, upregulation; green, downregulation. **(C)** Heat map of genes related to phenotypes of cells and mitochondria in *anti-3*, based on the RPKM values. **(D)** DEGs of *OX* (*OX* vs. WT) and their expression under H_2_O_2_ treatment (10 mM, 3 h) (corrected P-value < 0.001, fold change >1.5 or <–1.5; gray, genes recovered to normal expression level under H_2_O_2_ treatment). **(E)** Clustering displays of expression ratios (*OX* vs. WT). Red, upregulation; green, downregulation. **(F)** Heat map of genes related to phenotypes of cells and mitochondria in *OX*, based on RPKM values.

To further characterize the effects of *PtomtAPX* on mitochondria, we performed RNA-seq analyses of WT and *PtomtAPX*-antisense cells. Compared to the WT, 3,571 genes with significantly different expression levels (fold change >1.5 or < –1.5, and corrected P < 0.001) were detected in *anti-3*. (Fig. 7A and Supplemental Dataset 1). This indicates that oxidative damage is the cause of the significantly differential gene expression in *anti-3*.

We clustered genes with similar expression patterns to further investigate the dynamic trend of the normalization of gene expression. The 3,571 genes were grouped into six clusters (Fig. 7B and Supplemental Dataset 1). Genes in cluster 1 (2 genes) and cluster 2 (781 genes) were downregulated, and the genes in cluster 4 (18 genes) and cluster 5 (2,612 genes) were upregulated in *anti-3*, and were wholly or partly normalized in *anti-3*-A, indicating that they are directly related to the abnormal morphology of cells and mitochondria. The expression levels of genes in cluster 3 (83 genes) which were downregulated in *anti-3* and cluster 6 (75 genes) but upregulated in *anti-3* were not normalized in *anti-3*-A (Fig. 7B and Supplemental Dataset 1).

Gene Ontology (GO) term enrichment analyses were performed to further characterize the function of the differentially expressed genes in *anti-3* (Supplemental Dataset 2). The GO term “integral component of membrane” (P < 0.005) “UDP-glycosyltransferase activity” and “protein phosphorylation” (P < 0.005) showed enrichment (Supplemental Dataset 2). In particular, an OXPHOS-related gene (phosphatase family protein, POPTR_0007s09470) and inorganic pyrophosphatase (POPTR_0005s01840) were significantly downregulated in *anti-3* (Fig. 7C). The activity of inorganic pyrophosphatase, which hydrolyzes inorganic pyrophosphate into two phosphates, is essential for many biosynthetic reactions and energy metabolism as well as for maintaining mitochondrial function. The deletion of inorganic pyrophosphatase compromises cell viability due to the loss of mitochondrial function^31^. The decreased expression of those genes in *anti-3* was consistent with the damaged respiratory ETC in mitochondria of *anti-3*. The gene encoding phosphatidylinositol-4-phosphate 5-kinase (POPTR_0005s15850), which is involved in cytoskeletal rearrangement^32^, was also downregulated in *anti-3* (Fig. 7C). A cytokinin riboside 5′-monophosphate phosphoribohydrolase gene (POPTR_0004s22210), the product of which activates cytokinins^33^, and a set of UDP-glycosyltransferases (POPTR_0001s05400, POPTR_0006s02390, POPTR_0545s00210 and POPTR_0009s15190), which are related to mitosis and strongly induced in dividing cells^34^, were downregulated in *anti-3* (Fig. 7C). Cytokinins are master plant hormones that control cell division, senescence, and growth^35^. The decreased expression of those genes in *anti-3* is consistent with the reduced proliferation of transgenic cells. In addition, a gene related to leaf senescence, alkaline α-galactosidase (POPTR_0016s05500)^36^, was downregulated in *anti-3* (Fig. 7C). These genes are implicated in the altered growth and morphology of transgenic cells, which were smaller and had a slower growth rate than WT cells.

RNA-seq analyses of WT and *PtomtAPX*-overexpressed cells treated or not with 10 mM H_2_O_2_ were performed. Compared to the WT, 5,542 genes with significantly different expression levels (fold change >1.5 or <–1.5, and corrected P < 0.001) were detected in *OX*. The expression of 66.47% (3,684 genes) of those genes was normalized by 10 mM H_2_O_2_ (*OX*-H) (Fig. 7D and Supplemental Dataset 1). Thus, the decreased H_2_O_2_ content caused the significantly different gene expression levels in *OX*.

We also clustered genes with similar expression profiles to investigate the trends of these RNAs in *OX* compared to the WT. As shown in Fig. 7E, the 5,542 genes were grouped into six clusters (Supplemental Dataset 1). Genes in cluster 1 (1 genes) and cluster 2 (1,486 genes) were downregulated, and those in cluster 4 (127 genes) and cluster 5 (2,070 genes) were upregulated in *OX* and were normalized in *OX-*H, indicating that they are directly related to the abnormal morphology of cells and mitochondria. Genes in cluster 3 (643 genes), which were downregulated in *OX*, and cluster 6 (1,215 genes), which were upregulated in *OX-*H, were not normalized in *OX*-H (Fig. 7E and Supplemental Dataset 1). The GO term “hydrolase activity, acting on ester bonds” was significantly enriched (Supplemental Dataset 2). For example, two phosphatases (POPTR_0014s08130 and POPTR_0013s14030) were downregulated in *OX* and recovered to normal expression level in *OX*-H (Fig. 9F). Moreover, two cytokinin riboside 5′-monophosphate phosphoribohydrolases (POPTR_0016s09180 and POPTR_0009s01600), an UDP-glycosyltransferase (POPTR_0003s14230), and a phosphatidylinositol-4-phosphate 5-kinase (POPTR_0005s15850) were significantly differentially expressed in *OX*, and their expression was normalized in *OX*-H (Fig. 7F).The genes in cluster 5 were upregulated in *OX* and normalized in *OX*-H. For example, inorganic pyrophosphatase (POPTR_0006s08250) impairs mitochondrial function and was normalized in *OX*-H (Fig. 7F). These genes may be related to growth reduction, cell morphology and mitochondrial dysfunction due to their important roles in mitochondrial function, cytoskeleton, cytokinin activation, and cell division^31–34^. This is consistent with the need for the addition of 6-BA for the growth of *PtomtAPX*-overexpressed cells and the normalization of cellular morphology and growth rate, and mitochondrial function, by exogenous H_2_O_2_.

Non-enzymatic and enzymatic mechanisms maintain redox homeostasis in plant cells. H_2_O_2_ is scavenged principally by SOD, CAT, GPX, and PrxR, and the AsA-GSH cycle. None of these ROS-scavenging enzymes was significantly differentially expressed in *anti-3* or *OX* compared to the WT, suggesting that the ROS pathway is not induced by the downregulation or upregulation of *PtomtAPX*.

APX mediates cross-compartment protection against the deleterious effects of H_2_O_2_^37^. To determine whether APXs in other compartments can compensate for the loss of mitochondrial APX, we evaluated the expression levels of APXs in the chloroplasts, cytoplasm, and microbody. None of the APX genes was significantly differentially expressed in the *PtomtAPX*-antisense and *PtomtAPX*-overexpressed cell lines, suggesting that mitochondrial *PtomtAPX* is independent of APXs in other compartments and so provides little cross-compartment protection against H_2_O_2_.

Taken together, these results suggest that *PtomtAPX* expression is closely associated with H_2_O_2_ content and mitochondrial structure. Moreover, appropriate *PtomtAPX* expression is necessary to maintain a mitochondrial H_2_O_2_ level appropriate for mitochondrial structure and function in *P. tomentosa*.

## Discussion

Plant mitochondria are major organelles of ROS production and are targets of ROS^38,39^. ROS-scavenging systems control the ROS balance in plant mitochondria. We identified two APXs, *PtomtAPX* and *PtosAPX*, in the mitochondria of *P*. *tomentosa*. *PtomtAPX* is specifically targeted to mitochondria and is the major APX involved in controlling mitochondrial H_2_O_2_ levels, and it influences mitochondrial structure and function and PCD in *P*. *tomentosa*. *PtosAPX* is targeted to both mitochondria and chloroplasts and is the minor role in controlling mitochondrial H_2_O_2_ levels.

Targeting peptides of mitochondrial and chloroplastic proteins (mTPs, also termed pre-sequences and cTPs, also termed transit peptides) has a similar amino acid composition and are indistinguishable by sequence analyses^40,41^. It is possible that mitochondrial isoforms have been misidentified as chloroplastic isoforms. For example, there were four chloroplastic APX in rice, but no mitochondrial isoform was confirmed^2,5,6^, indicating that mitochondrial isoform was mistaken for chloroplastic iosform. In this study, *PtomtAPX* and *PtosAPX* of *P*. *tomentosa* were also marked as chloroplastic isoforms (Supplemental Fig. 2). However, plant mTPs and cTPs exhibit quantitative and structural differences^42^.

Positively charged residues and amphipathic α-helix formed in 19 N-terminal amino acid are responsible for localization^26,42,43^. Our results indicate that chloroplastic APX has high serine and proline contents and a low arginine content. By contrast, *PtomtAPX* has three arginine residues and two amphipathic α-helices in its 19 N-terminal portion of targeting peptide (Fig. 1A). A rice chloroplastic APX isoform, OsAPX6, has been found in mitochondria of BY-2 tobacco but its presence in chloroplasts is unclear^2,6,44^. We found that the mTPs of OsAPX5 and OsAPX6 contained four positively charged amino acids (three of them are arginine residues) and an amphipathic α-helix, suggesting that they are targeted to mitochondria (Fig. 1A). Furthermore, three positively charged amino acids (two of them are arginine residues) and an amphipathic α-helix are present in *Z*. *mays* APX7 (Fig. 1A). Therefore, mitochondrial APX isoforms are present in various plant species, including model plants such as rice. The results of comparing the amino acid composition of targeting peptide of at least 100 proteins from diverse plant species showed increased intermediate amino acid led to the targeted of both chloroplasts and mitochondria^44–48^. Targeting of the same protein to two locations can result in co-regulation without a change in the genome. The presence of co-targeted enzymes of the AsA-GSH cycle may have a beneficial effect by restoring a normal level of ROS, which is important for the function of mitochondria and plastids.

Plant mitochondria in photosynthetic and non-photosynthetic organs have defense systems to prevent damage by H_2_O_2_ and to respond to environmental stresses^49–51^. Our results indicate that *P*. *tomentosa* has at least two mitochondrial APXs, *PtomtAPX* and *PtosAPX*, which have similar levels of activity. However, mitochondria had a low *PtosAPX* level, suggesting that *PtosAPX* is not an important player in ROS scavenging in mitochondria. The *PtosAPX* level in mitochondria was markedly lower than that of *PtomtAPX*, while the *PtosAPX* level in chloroplasts was higher than that in mitochondria, suggesting that *PtomtAPX* and *PtosAPX* are mitochondrial and chloroplastic isoforms, respectively. This is consistent with a previous report that dual-targeted proteins are more strongly targeted to one of the two organelles^44^.

The expression of *PtomtAPX* and *PtosAPX* differed significantly in the presence of various environmental stresses. In this study, the expression of *PtosAPX* and *PtotAPX* increased by high-light stress, whereas that of *PtomtAPX* was only slightly affected. The expression of chloroplastic APX(chlAPX) varies according to light intensity; e.g., high-intensity light decreases the expression of chlAPX in spinach leaves^52^. Exposure of *Arabidopsis* lacking tAPX to high-intensity light and MV stresses result in increased H_2_O_2_ accumulation and oxidation of proteins^53^. A mutant wheat line with decreased tAPX activity shows reduced photosynthetic activity and biomass accumulation in the presence of high-intensity light, suggesting that tAPX is essential for photosynthesis. Therefore, *PtosAPX* plays a role in the photosynthetic apparatus and *PtomtAPX* functions in the non-photosynthetic apparatus.

The major H_2_O_2_ detoxifying system in plant cells is the AsA-GSH cycle, in which APX is the key enzyme^49,50^. However, stromal/mitochondrial APX-knockdown lines, as well as single and double null mutants in chlAPX (sAPX and tAPX) in *A*. *thaliana* and rice, exhibit a normal phenotype and normal biochemical and physiological attributes under normal growth conditions, suggesting that chloroplast stromal/mitochondrial APX is not important for H_2_O_2_ scavenging in *A*. *thaliana* or rice^23,24^. In our research, the *PtomtAPX*-antisense cells under normal growth conditions had significantly higher H_2_O_2_ levels and an abnormal phenotype, including impaired mitochondrial structure and function and slowed growth. Transcriptomic analyses of *anti-3* and WT revealed that the expression levels of genes encoding phosphatases and inorganic pyrophosphatases were significantly different, while genes related to cell growth (such as cytoskeleton, cytokinin, cell division, and senescence) were downregulated in *anti-3*. *PtosAPX*, was not upregulated in *anti-3*, indicating that *PtosAPX* cannot compensate for the function of *PtomtAPX*. Therefore, *PtomtAPX*, not *PtosAPX*, is the major APX in mitochondria.

Glutathione acts as a mobile pool of non-protein reduced sulfur, as an antioxidant, and in the detoxification of xenobiotics and heavy metals^1,30^. The GSH:GSSG ratio is an important indicator of the redox balance in plant cells. GR plays a central role in maintaining the GSH pool during stress^1^. The transcript level of *GR*, which plays a central role in maintaining the GSH pool, was not significantly increased in the *PtomtAPX-antisense* cells, and the GSH/GSSG ratio was significantly decreased, suggesting an insufficient glutathione pool to counteract the H_2_O_2_ level. Moreover, none of the APXs were significantly differentially expressed in *anti-3* or *OX* according to RNA-seq analyses, indicating that cross-compartment protection among different organelles is not enough to protect mitochondria in the absence of *PtomtAPX*. Therefore, *PtomtAPX* is required to modulate H_2_O_2_ levels in mitochondria.

Over-accumulation of ROS triggers plant cell death. Excess ROS alter mitochondrial membrane permeability, decrease ΔΨm, reduce the electron density and number of cristae, and compromise inner and outer membranes; these effects trigger the release of cytochrome c, resulting in PCD. In plants, PCD is activated during various developmental processes and under diverse stress conditions^54,55^. Our results indicate that PCD was triggered by increased H_2_O_2_ levels and decreased *PtomtAPX* expression in the *PtomtAPX*-antisense cells. The addition of exogenous H_2_O_2_ to the *PtomtAPX*-antisense cells resulted in increased PCD. This is consistent with the slowed plant growth, small cells, and mitochondrial aggregation, swelling, loss of electron density and cristae, and decrease in ΔΨm in the *PtomtAPX*-antisense lines. Thus, downregulation of *PtomtAPX* expression in the *PtomtAPX*-antisense plants induced PCD and slowed growth. This may explain the difficulty generating *PtomtAPX*-antisense plants under normal growth conditions.

The decreased levels of *PtomtAPX* protein in the *PtomtAPX*-antisense cells resulted in higher H_2_O_2_ levels, which could not be counteracted by enzymatic or non-enzymatic mechanisms due to damaged and dysfunctional mitochondria. ROS can damage, by indiscriminate oxidation of macromolecules, various cellular compartments. In this study, excessive H_2_O_2_ levels impaired mitochondrial function in the transgenic lines. This is consistent with a previous report that high levels of ROS oxidize mitochondrial structural proteins, which reduces the efficiency of OXPHOS^56^. The damage to mitochondria was significantly alleviated by the addition of exogenous AsA.

The decreased H_2_O_2_ levels in the *PtomtAPX*-overexpressed cells resulted in decreased membrane potential and ATP production. However, mitochondrial lipids and structural proteins, specifically those related to OXPHOS, were not oxidized, suggesting that maintenance of ROS homeostasis is necessary for OXPHOS in the *PtomtAPX*-overexpressed cells. Moreover, RNA-seq showed that the expression of genes related to mitochondrial function, cytoskeleton, cytokinin activation, and cell division in *OX* cells was significantly recovered by the addition of exogenous H_2_O_2_. Thus, low H_2_O_2_ levels also impair mitochondrial function by an unknown mechanism.

ROS as signaling molecules regulate a number of processes during plant growth and development, such as cell elongation and differentiation, as well as the responses to a variety of environmental stimuli^56,57^. For example, in soybean, H_2_O_2_ levels increase from the tip of the hypocotyl (elongation zone) to the highly lignified base. In onion root fragments, H_2_O_2_ levels are high in cells undergoing elongation and lignification, and the former have lower total peroxidase activity^58^. Therefore, maintaining appropriate H_2_O_2_ levels is important for mitochondrial efficiency and cell growth in *P*. *tomentosa*, although the mechanism involved is unknown.

In conclusion, *PtomtAPX* is specifically targeted to mitochondria in which it is the primary APX, while *PtosAPX* is dual-targeted to mitochondria and chloroplasts. Expression of *PtomtAPX* in mitochondria is necessary for regulation of mitochondrial ROS levels and maintenance of mitochondrial function in *P*. *tomentosa*. Our findings provide valuable insight into the mechanism by which ROS balance is maintained and the function of the AsA-GSH cycle in mitochondria.

## Materials and Methods

### Plant materials and growth conditions

Suspension cells of *P*. *tomentosa* Carr. were grown in the dark at 25 °C. Transgenic suspension cells were obtained according to Li *et al*.^59^.

### Molecular cloning and plasmid construction

Full-length *PtomtAPX* and *PtosAPX* cDNA were amplified using the primers *PtomtAPX-F/R* and *PtosAPX-F/R*, respectively, and sequenced. *PtomtAPX* and *PtosAPX* was amplified using *G-PtomtAPX-F/R* and *G-PtosAPX-F/R* and cloned into the pBI121-GFP vector to generate *35S:PtomtAPX-GFP* and *35S:PtosAPX-GFP*, respectively. Full-length *PtomtAPX* cDNA was amplified using *OX-PtomtAPX-F/R* and cloned into pBI121 to generate *35S:PtomtAPX* (Supplemental Table 1). Full-length *PtomtAPX* cDNA was amplified using *anti-PtomtAPX-F/R* and cloned into pBI121. The amphiphilic α-helice is predicted using Bioedit 7.0.

### Enzyme-activity assays

The ORF minus the targeting peptide of *PtomtAPX* and *PtosAPX* was amplified by PCR using *P-PtomtAPX-F/R* and *P-PtostAPX-F/R*, respectively, and inserted downstream of the pET30a plasmid T7 promoter (Novagen, Madison, WI). *PtomtAPX* and *PtosAPX* were expressed and purified according to Zhang *et al*.^60^. The activity of purified recombinant *PtomtAPX* using ASA and H_2_O_2_ as substrates was assayed as described previously^59^.

### *PtomtAPX* and *PtosAPX* immunolocalization

Purified recombinant *PtomtAPX* or *PtosAPX* (5 mg) was used to immunize rabbits at 3-week intervals. The specificity of the anti-PtomtAPX and anti-PtosAPX antibodies was confirmed by hybridization with a membrane blotted with protein extracts from leaves of *P*. *tomentosa*. *P*. *tomentosa* leaves were fixed and subjected to immunolocalization as described previously^61^.

### PtomtAPX-GFP and PtosAPX-GFP fusion targeting analyses

The *GFP* gene was amplified by PCR using the primers *GFP-F/GFP-R*. The product was inserted into pBI121(Clontech), which lacked the GUS-coding region to create the GFP(A)-pBI121 vector, the fusion construct of *35S:PtomtAPX-GFP*, and *35S:PtomtAPX-GFP*. Transgenic tobacco leaf epidermis and root-tip cells were visualized by CLSM (Leica SP8).

### Enzyme-linked immunosorbent assays (ELISA)

An anti-PtomtAPX or anti-PtosAPX antibody (1:5,000 in PBS) was added to each well of a microtiter plate, followed by a goat anti-rabbit IgG HRP-conjugated secondary antibody (1:10,000 in PBS) (Supplemental Fig. 6). Protein from leaves or suspension cells was used for ELISA in accordance with the method of Zhang *et al*.^61^. Optical density values were determined using a Bio-Rad 680 Microplate Reader.

### qRT-PCR

qRT-PCR was performed with the *qRT-PtomtAPX-F/R* or qRT-PtosAPX primers. The PCR conditions were 94 °C for 5 min; 40 cycles at 94 °C for 20 s, 58 °C for 20 s, 72 °C for 30 s, and 60 °C for 30 s; and 72 °C for 1 min. qRT-actin-F/R was used as normalization. Data were calculated using the 2^−ΔΔCt^ method.

### TEM

Suspension cells were fixed and embedded as described by Zhang *et al*.^61^.

### CLSM

CLSM was performed using a Leica TCS-SP8 CLSM. The JC-1 signal was visualized by excitation at 488 nm and emission at 520–540 nm (green fluorescence) and 595–625 nm (red fluorescence). MitoTracker Red CMXRos (Invitrogen) was visualized by excitation at 578 nm and emission at 580–620 nm. Quantification was performed using Leica confocal software.

### Staining

Suspension cells and root-tip cells of *P*. *tomentosa* were incubated in 0.3 μM CMXRos in slice buffer (0.3 mM sucrose, 5 mM TES, 5 mM MgCl_2_) at 30 °C in the dark for 10 min as described by Lord *et al*.^46^. Suspension cells and root-tip cells of *P*. *tomentosa* were incubated in 100 μM JC-1 for 20 min as described by Wang *et al*.^62^.

### Isolation of mitochondria and chloroplast

Mitochondria or chloroplasts were isolated from leaves of *P*. *tomentosa*. The purity of the preparations was detected using cytochrome c oxidase for mitochondria, catalase for peroxisomes, alcohol dehydrogenase for cytosol, and alkaline pyrophosphatase for plastids (Supplemental Fig. 7) as described previously^63^. The chloroplast fractions were enriched using Minute^TM^ chloroplast isolation kit(Invent Biotechnologies).

### Determination of H_2_O_2_ and MDA contents, ASA/DHA, GSH/GSSG, and ATP/ADP ratios, and ETC complex activity

H_2_O_2_ content was detected using Fluorimetric Hydrogen Peroxide Assay Kit (Sigma-Aldrich). MDA content was detected using Lipid Peroxidation (MDA) Assay Kit (Sigma-Aldrich). ADP and ATP levels were detected using a EnzyLight™ ATP assay (BioAssay Systems).The levels of AsA, GSSG and GSH were detected as described previously^4,64^.

### Immunoblotting

The appearance of carbonyl groups in proteins was detected using an OxiSelect™ Protein Carbonyl Immunoblot Kit (Cell Biolabs, Inc.).

### Flow cytometry

Suspension cells of *P*. *tomentosa* were incubation for 30 min in the dark with binding buffer (10 mM HEPES [pH 7.4], 140 mM NaCl, 1 mM MgCl_2_, 5 mM KCl, 2.5 mM CaCl_2_,1 μg/mL Hoechst and 1 μg/mL PI) and analyzed on a FACSCalibur flow cytometer (BD Biosciences).

### RNA-Seq sample collection, Illumina sequencing, and data processing

Total RNA was isolated from WT, *anti-3*, and *OX* cells using TRIzol reagent according to the manufacturer’s protocol (Invitrogen). The samples were sequenced using an Illumina Genome Analyzer (HiSeq™ 2000; Illumina, San Diego, CA). The raw reads were data-filtered to obtain high-quality clean reads. The clean reads were mapped to the *P*. *trichocarpa* reference genome and reference genes using SOAPaligner/SOAP2. No more than two mismatches were allowed in the alignment. Gene expression levels were calculated as reads per kilobase per million reads (RPKM)^65^. The data are deposited in the National Center for Biotechnology Information Gene Expression Omnibus database (https://www.ncbi.nlm.nih.gov/geo/query/acc.cgi?acc=GSE121562) under accession number GSE121562.

### Identification and analysis of differentially expressed genes

Differentially expressed genes (DEGs) among WT, *anti-3*, and *OX* cells were identified using the DEGseq R package (1.12.0)^65^ based on the normalized read counts. A corrected P-value < 0.001 and |log2ratio| > 1.5 were the thresholds for significantly different expression. GO enrichment analyses of the DEGs were performed using the GOseq R package^66^. GO terms with a P-value < 0.005 were considered significantly enriched in DEGs.

### Accession numbers

Sequence data from this article can be found in the NCBI database under accession numbers MH910690 (*PtomtAPX*) and MH910610 (PtosAPX), AB022273 (Nt.tAPX), AB114856 (Os.tAPX), D77997 (So.tAPX), D83656 (Cka.tAPX), AB022274 (Nt.sAPX), AB114855 (Os.sAPX), D83669 (So.sAPX) and D88420 (Cka.sAPX).

## Supplementary information

Supplementary Information

Supplemental Dataset 1

Supplemental Dataset 2
