# Supplementary material for: PtomtAPX, a mitochondrial ascorbate peroxidase, plays an important role in maintaining the redox balance of Populus tomentosa Carr
Source: Sci Rep. 2019 Dec 20;9:19541. doi: 10.1038/s41598-019-56148-w (PMC6925217; doi:10.1038/s41598-019-56148-w)
Supplement: Supplementary file 1 — Supplementary Information [file 41598_2019_56148_MOESM1_ESM.pdf]

***PtomtAPX*, a mitochondrial ascorbate peroxidase, plays an important role in maintaining the redox balance of *Populus tomentosa* Carr.**

Bin Yin[a,b,1], Jiaxue Zhang[b,1], Yadi Liu[b,1], Xiang Pan[b,1], Zhijing Zhao[b], Hui Li[b], Chong Zhang[b], Conghui Li[b], Xihua Du[b], Yinjun Li[b], Di Liu[b,2], Hai Lu [a,b,2]

[a]Beijing Advanced Innovation Center for Tree Breeding by Molecular Design, Beijing Forestry University, Beijing 100083, People's Republic of China

[b]College of Biological Sciences and Biotechnology, Beijing Forestry University, Beijing 100083, People's Republic of China

[1]These authors contributed equally to this work

[2] Correspondence Author: liudi@bjfu.edu.cn; luhai1974@bjfu.edu.cn

Supplementary Information

The following materials are available in the online version of this article.

Supplemental Figure 1. Nucleotide and deduced amino acid sequences of *PtomtAPX* and *PtosAPX*. Box, signal peptide sequence; arrow, cleavage site; asterisk, stop codon.

Supplemental Figure 2. Phylogenetic tree of APXs.

Sequences of APXs from *Arabidopsis thaliana*, *Oryza sativa*, *Zea mays*, *Selaginella moellendorffii*, *Chlamydomonas reinhardtii*, *Volvox carteri*, *Coccomyxa subellipsoidea*, *Ostreococcus lucimarinus*, *Micromonas pusilla*, *Brassica rapa*, *Glycine max*, *Populus trichocarpa*, and *Populus tomentosa* were used. The phylogenetic tree was generated by the neighbor-joining method with 1,000 bootstraps using MEGA 6. The rectangular box shows the position of *PtomtAPX* and *PtosAPX*.

Supplemental Figure 3. Localization of ROS in mitochondria of various cell lines stained using CMXRos and H2DCFDA.

(A,D,G,J,M) Mitochondria stained using CMXRos. (B,E,H,K,N) Mitochondria stained using H2DCFDA. (C,F,I,L,O) Merged images. Bars, 10 μm.

Supplemental Figure 4. Mitochondrial oxidant levels.

(A) ASA:DHA ratio. **(B)** GSH:GSSG ratio. **(C)** MDA content. **(D)** Carbonylation of mitochondrial proteins. ******significantly different at $P < 0.01$. Bars, standard deviations.

Supplemental Figure 5. Mitochondrial morphology and activity in *PtomtAPX*-overexpressed cells under H_2O_2 and AsA treatment.

(A) Mitochondrial H_2O_2 content under H_2O_2 treatment. **(B)** Mitochondrial ATP:ADP ratio under H_2O_2 treatment. **(C)** Frequencies of the types of mitochondria under H_2O_2 treatment. **(D)** $\Delta\psi m$ of mitochondria under H_2O_2 treatment (JC-1 staining). Frequencies and ratios were calculated based on 200 cells. WT, untreated WT cells; *OX*, untreated *PtomtAPX*-overexpressed cells; *OX*-10, 10 mM H_2O_2 treatment; *OX*-100, 100 mM H_2O_2 treatment. ******significantly different at $P < 0.01$. Bars, standard

deviations.

Supplemental Figure 6 (A) Full-length blot of anti-PtomtAPX antibodies.**(B**) Full-length blot of anti-PtosAPX antibodies.**(C)** Full-length blot of Carbonylation of mitochondrial proteins

Supplemental Figure 7. Isolation of mitochondria by density centrifugation.

(A) The organelle pellet from *P. tomentosa* leaves or homogenized cells was loaded onto a Percoll step gradient consisting of steps of 40% (fractions 27–30), 23% (fractions 10–26), and 18% Percoll (fractions 1–9). After centrifugation, mitochondria were recovered from the 40%:23% interface (fractions 18–30). **(B)** recovered mitochondria were loaded onto a self-forming Percoll gradient containing 28% Percoll. Fractions (1 mL) were collected from both gradients (from top to bottom) and analyzed for the activities of marker enzymes of mitochondria.

Supplemental Table 1. Primers used for molecular cloning, plasmid construction, and qRT-PCR analyses.

Supplemental Dataset 1. Significant up regulation or down regulation of differentially expressed genes (DEGs) (P-value <0.001, fold change >1.5 or <-1.5) in *anti-3*; DEGs in *OX* and their expression in *OX*-H, both comparing with WT. WT, untreated WT; *OX*, untreated *PtomtAPX*-overexpressed cells; *anti-3*, untreated *PtomtAPX*-antisense cells; *OX*-H, *PtomtAPX*-overexpressed cells treated with 10 mM H_2O_2.

Supplemental Dataset 2. Gene ontology (GO) term enrichment of differentially expressed genes (DEGs) in *anti-3* and *OX*.

GO terms that are significantly enriched (P-value < 0.005) in cluster 1 to cluster 6. WT, untreated WT; *OX*, untreated *PtomtAPX*-overexpressed cells; *anti-3*, untreated *PtomtAPX*-antisense cells.

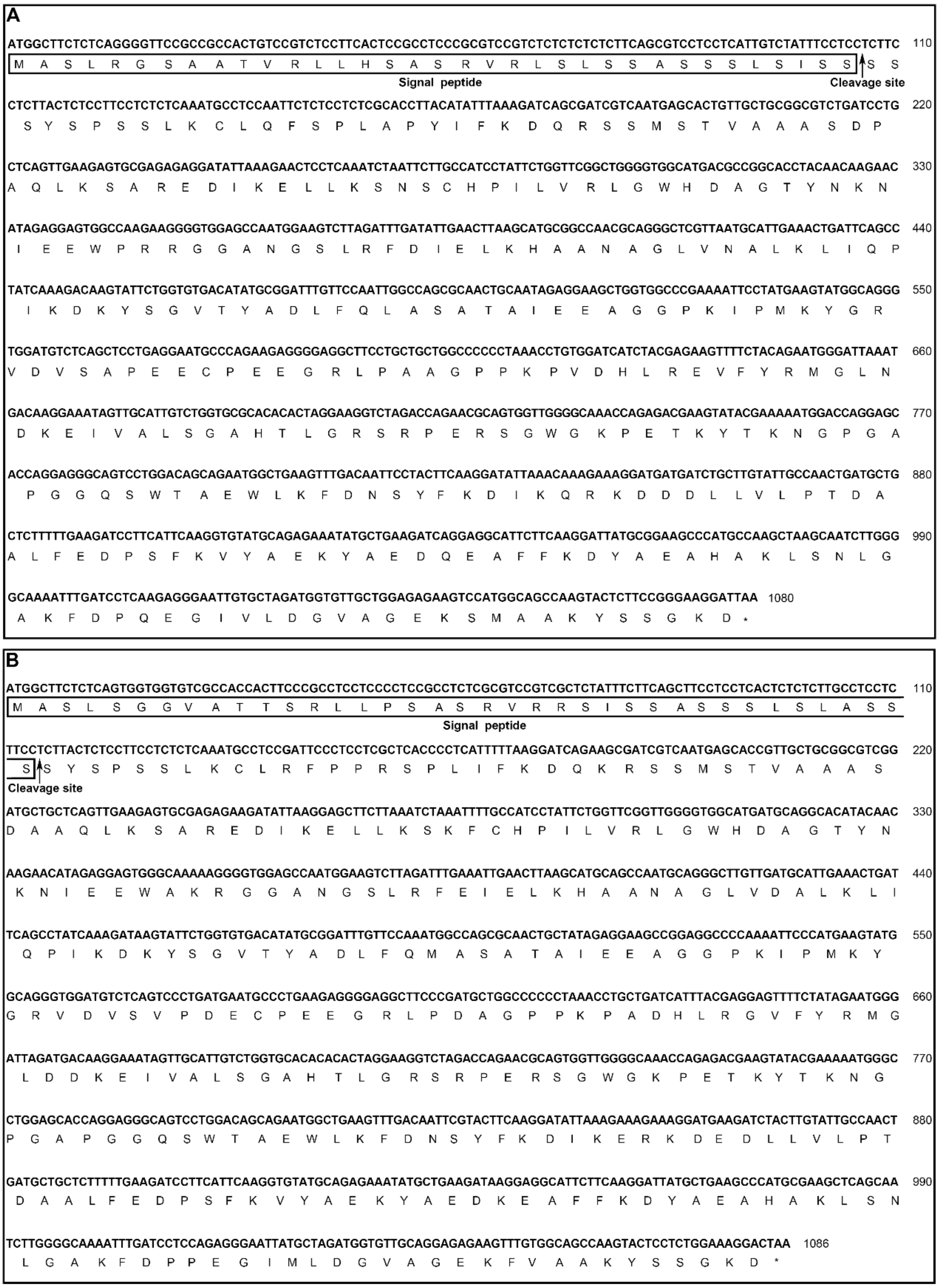

Supplemental Figure 1. Nucleotide and deduced amino acid sequences of *PtomtAPX* and *PtosAPX*. Box, signal peptide sequence; arrow, cleavage site; asterisk, stop codon.

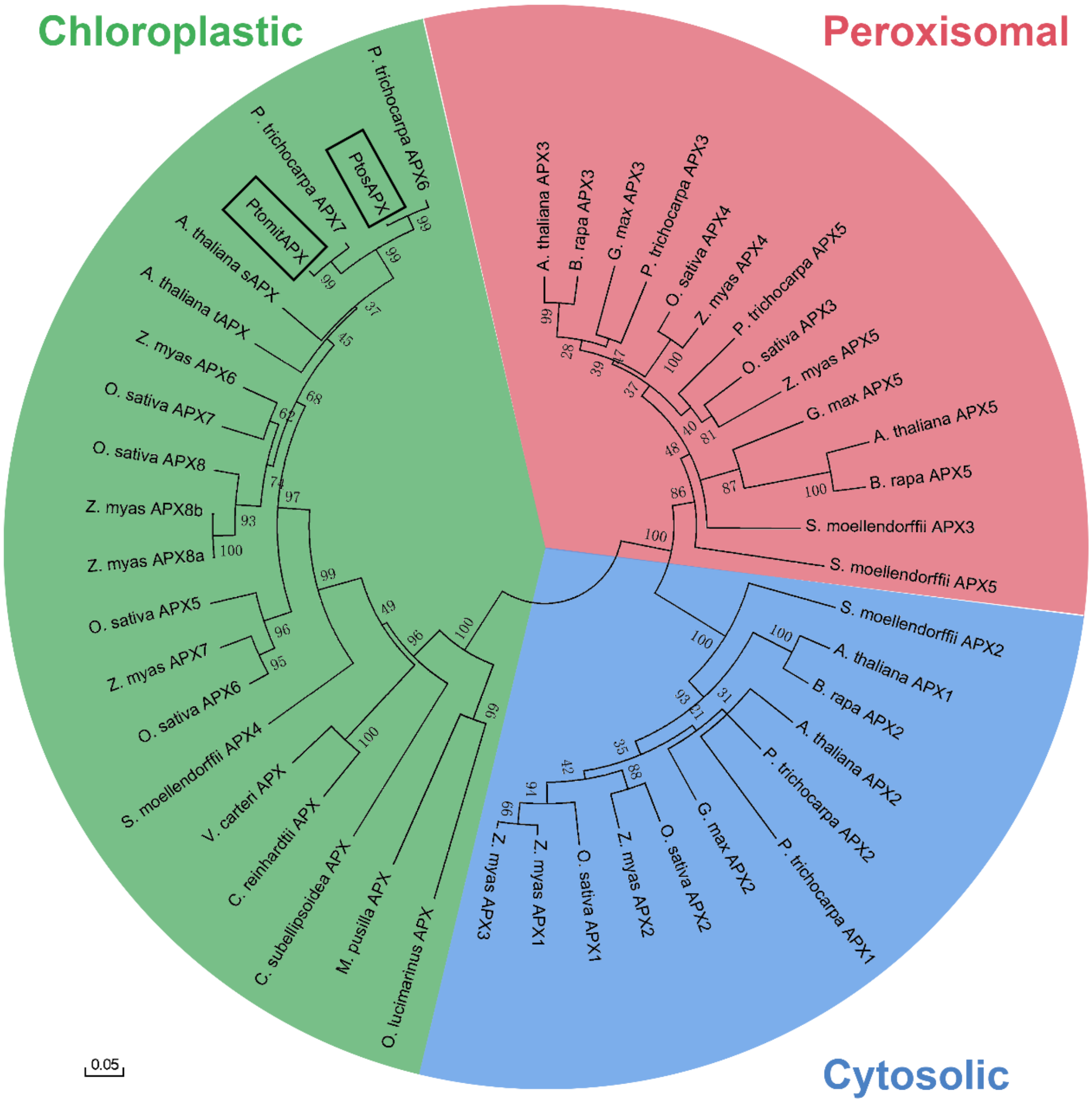

Supplemental Figure 2. Phylogenetic tree of APXs.

Sequences of APXs from *Arabidopsis thaliana*, *Oryza sativa*, *Zea mays*, *Selaginella moellendorffii*, *Chlamydomonas reinhardtii*, *Volvox carteri*, *Coccomyxa subellipsoidea*, *Ostreococcus lucimarinus*, *Micromonas pusilla*, *Brassica rapa*, *Glycine max*, *Populus trichocarpa*, and *Populus tomentosa* were used. The phylogenetic tree was generated by the neighbor-joining method with 1,000 bootstraps using MEGA 6. The rectangular box shows the position of *PtomtAPX* and *PtosAPX*.

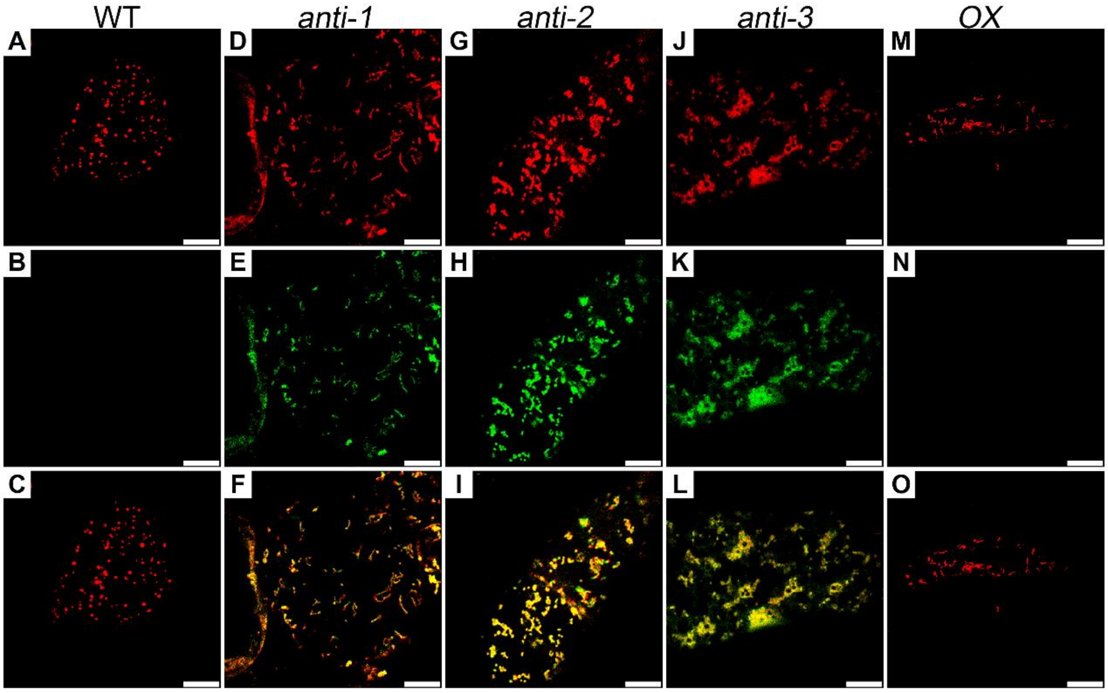

Supplemental Figure 3. Localization of ROS in mitochondria of various cell lines stained using CMXRos and H_2DCFDA.

(A,D,G,J,M) Mitochondria stained using CMXRos. **(B,E,H,K,N)** Mitochondria stained using H_2DCFDA. **(C,F,I,L,O)** Merged images. Bars, 10 μm.

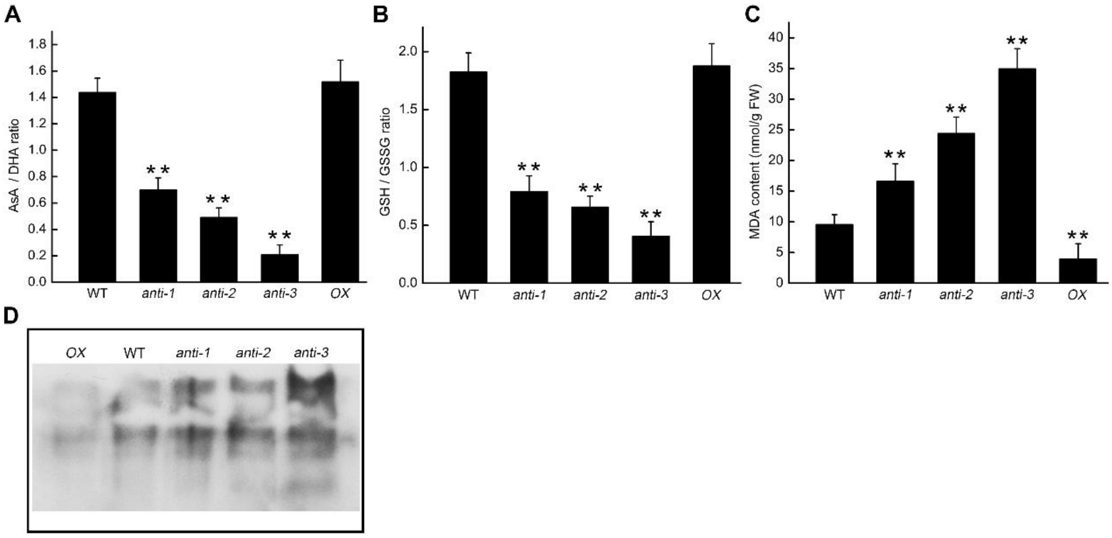

Supplemental Figure 4. Mitochondrial oxidant levels.

(A) AsA:DHA ratio. **(B)** GSH:GSSG ratio. **(C)** MDA content. **(D)** Carbonylation of mitochondrial proteins. ****significantly different at $P < 0.01$. Bars, standard deviations.

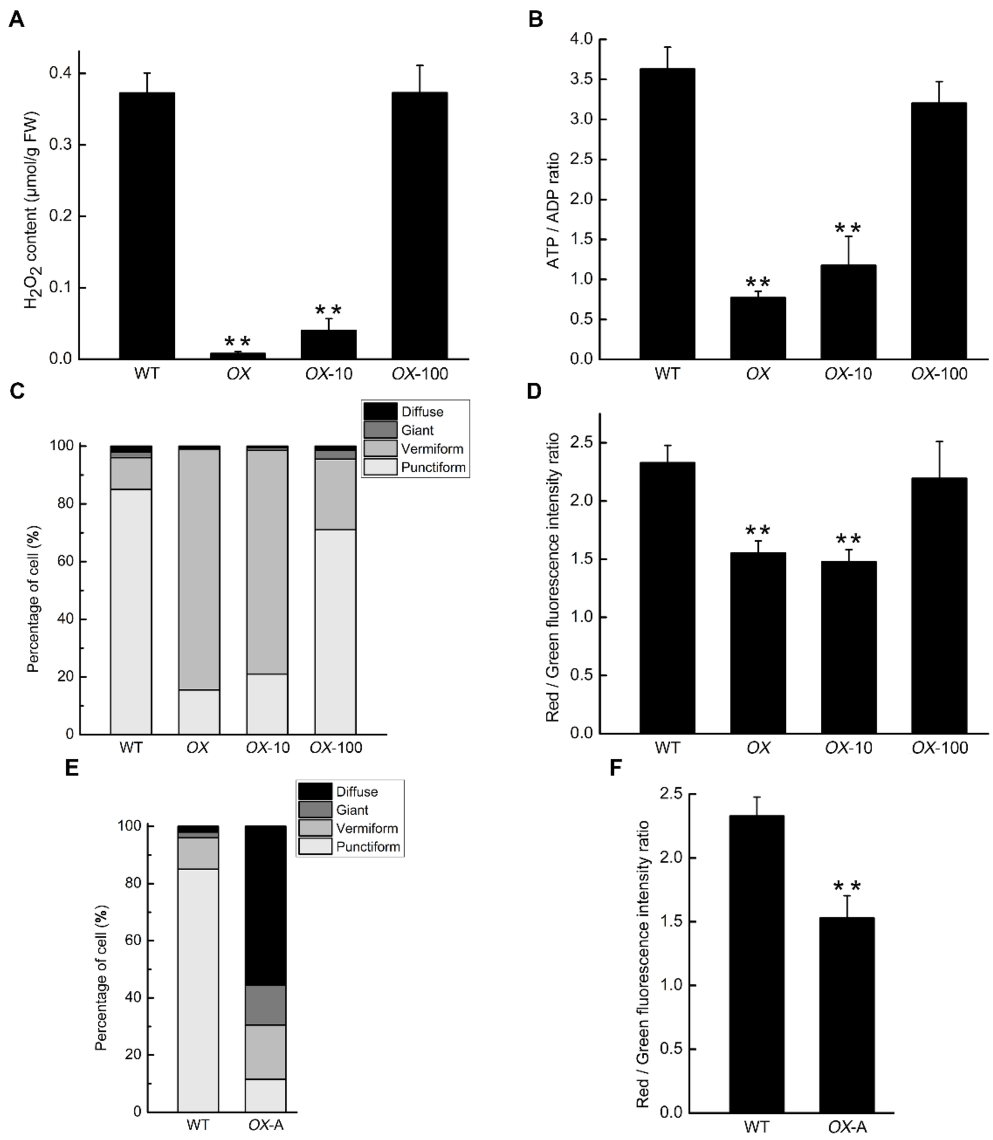

Supplemental Figure 5. Mitochondrial morphology and activity in *PtomtAPX*-overexpressed cells under H_2O_2 and AsA treatment.

(A) Mitochondrial H_2O_2 content under H_2O_2 treatment. **(B)** Mitochondrial ATP:ADP ratio under H_2O_2 treatment. **(C)** Frequencies of the types of mitochondria under H_2O_2 treatment. **(D)** Δψm of mitochondria under H_2O_2 treatment (JC-1 staining). **(E)** Frequencies of the types of mitochondria under AsA treatment. **(F)** Δψm of mitochondria under AsA treatment (JC-1 staining). Frequencies and ratios were calculated based on 200 cells. WT, untreated WT cells; *OX*, untreated *PtomtAPX*-overexpressed cells; *OX*-10, 10 mM H_2O_2 treatment; *OX*-100, 100 mM H_2O_2 treatment; and *OX*-A, 1 mM AsA treatment. **significantly different at $P < 0.01$. Bars, standard deviations.

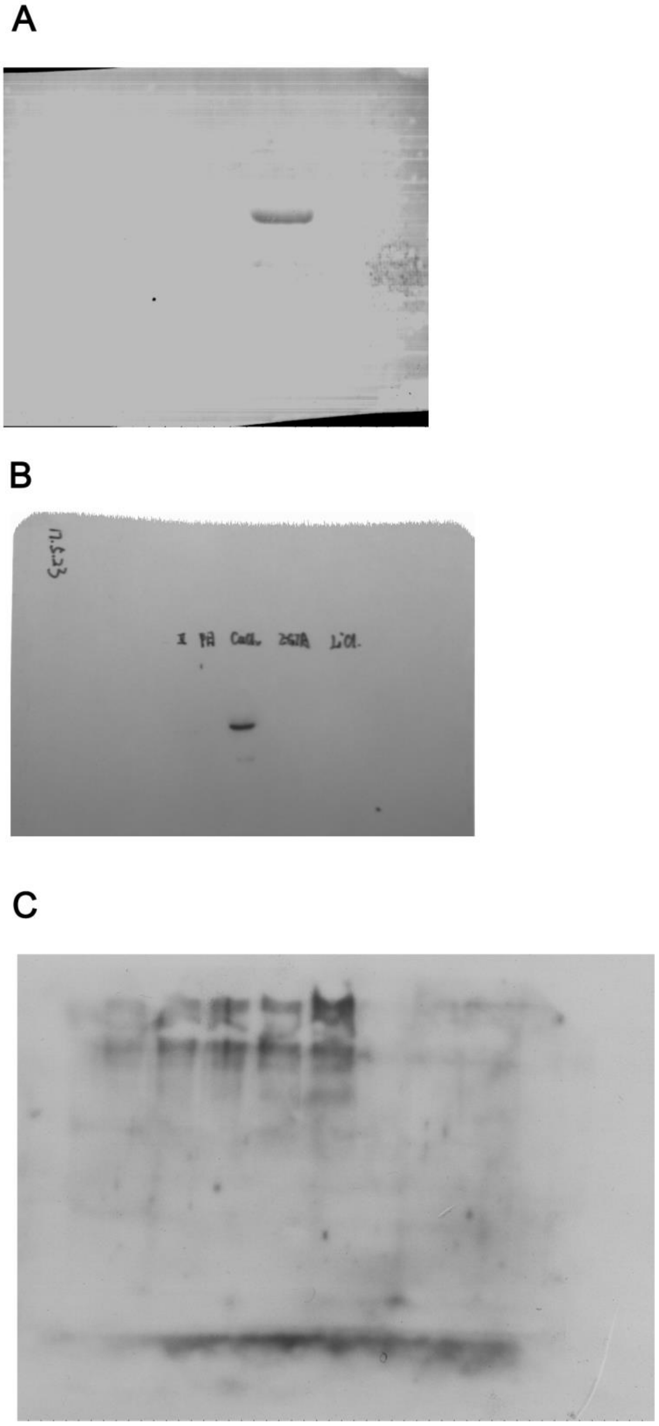

Supplemental Figure 6.(A) Full-length blot of anti-PtomtAPX antibodies.(B) Full-length blot of anti-PtosAPX antibodies.(C) Full-length blot of Carbonylation of mitochondrial proteins

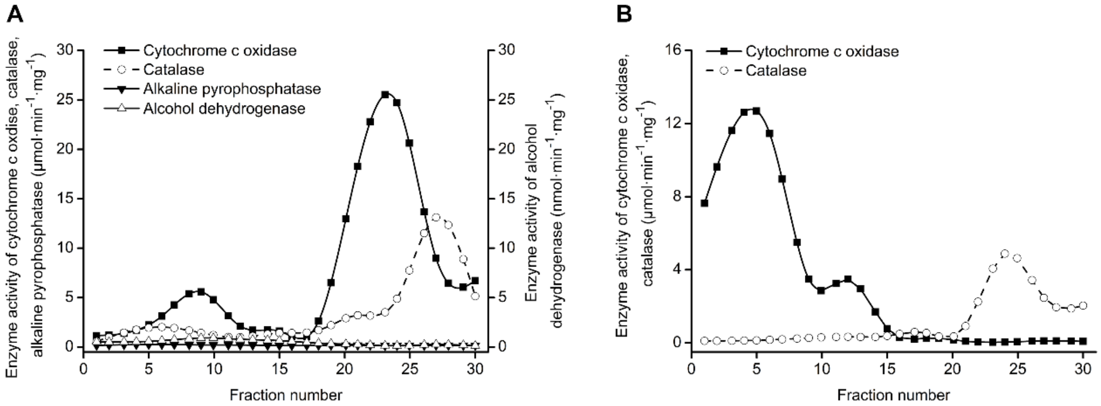

Supplemental Figure 7. Isolation of mitochondria by density centrifugation.

(A) The organelle pellet from *P. tomentosa* leaves or homogenized cells was loaded onto a Percoll step gradient consisting of steps of 40% (fractions 27–30), 23% (fractions 10–26), and 18% Percoll (fractions 1–9). After centrifugation, mitochondria were recovered from the 40%:23% interface (fractions 18–30). **(B)** recovered mitochondria were loaded onto a self-forming Percoll gradient containing 28% Percoll. Fractions (1 mL) were collected from both gradients (from top to bottom) and analyzed for the activities of marker enzymes of mitochondria.

Supplemental Table 1. Primers used for molecular cloning, plasmid construction, and qRT-PCR analyses.

Primers	Primer sequences（5′-3′）	Restriction sites
PtomtAPX-F	CGCAACCAATGGCTTCTCTCAG	N/A
PtomtAPX-R	ACACCGCAATTAAAGCCAAGTG	N/A
PtosAPX-F	ATGGCTTCTCTCAGTGGTG	N/A
PtosAPX-R	GTCCTTTCCAGAGGAGTACTTG	N/A
G-PtomtAPX-F	CCGCTCGAGATGGCTTCTCT	*Xho* I
G-PtomtAPX-R	GGACTAGTATCCTTCCCGGA	*Spe* I
G-PtosAPX-F	CCGCTCGAGATGGCTTCTCT	*Xho* I
G-PtosAPX-R	GGACTAGTATTTCCAAGAAGAGATG	*Spe* I
OX-PtomtAPX-F	CGGGATCCATGGCTTCTCTCAGGGGTTCC	*BamH* I
OX-PtomtAPX-R	CGAGCTCTTAATCCTTCCCGGAAGAGTA	*Sac* I
anti-PtomtAPX-F	CGAGCTCATGGCTTCTCTCAGGGGTTCC	*Sac* I
anti-PtomtAPX-R	CGGGATCCTTAATCCTTCCCGGAAGAGTA	*BamH* I
P-PtomtAPX-F	CGGGATCCTACTCTCCTTCCTCTCTCA	*BamH* I
P-PtomtAPX-R	CCCAAGCTTTTAATCCTTCCCGGAAGAG	*Hind* III
P-PtosAPX-F	CGGGATCC ATGGCTTCTCTCAGTGGTG	*BamH* I
P-PtosAPX-R	CCCAAGCTTGTCCTTTCCAGAGGAGTACTTG	*Hind* III
qRT-actin-F	AAACTGTAATGGTCCTCCCTCCG	N/A
qRT-actin-R	GCATCATCACAATCACTCTCCGA	N/A
qRT-PtomtAPX-F	CTGGAAAGAGAGAGTTGTCAG	N/A
qRT-PtomtAPX-R	GTGCCAGAACAGCAATCAC	N/A
qRT-PtosAPX-F	CATCCTATTCTGGTTCGGTTG	N/A
qRT-PtosAPX-R	TTGGCTGCATGCTTAAGTTC	N/A
qRT-PtotAPX-F	CTGGAAAGAGAGAGTTGTCAG	N/A
qRT-PtotAPX-R	GTGCCAGAACAGCAATCAC	N/A
